# Fully Complementary Higher Dimensional Partitions

**DOI:** 10.1007/s00026-024-00691-5

**Published:** 2024-04-06

**Authors:** Florian Schreier-Aigner

**Affiliations:** University of Vienna, Wien, Austria

**Keywords:** Plane partitions, Higher dimensional partitions, MacMahon, Symmetry classes

## Abstract

We introduce a symmetry class for higher dimensional partitions—*fully complementary higher dimensional partitions* (FCPs)—and prove a formula for their generating function. By studying symmetry classes of FCPs in dimension 2, we define variations of the classical symmetry classes for plane partitions. As a by-product, we obtain conjectures for three new symmetry classes of plane partitions and prove that another new symmetry class, namely *quasi-transpose-complementary plane partitions*, are equinumerous to symmetric plane partitions.

## Introduction

**Fig. 1:**
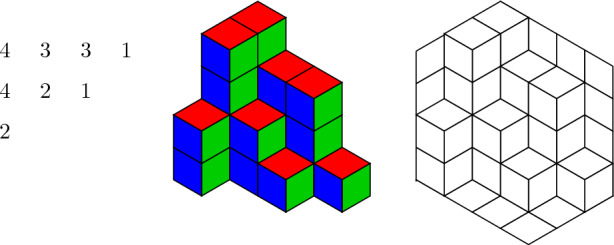
A plane partition contained in a (3, 4, 4)-box on the left, its graphical representation as stacks of unit cubes (middle) and the associated lozenge tiling (right)

A *plane partition* $\pi$ is an array $(\pi _{i,j})$ of non-negative integers with all but finitely many entries equal to 0, which is weakly decreasing along rows and columns, i.e. $\pi _{i,j} \ge \pi _{i+1,j}$ and $\pi _{i,j} \ge \pi _{i,j+1}$; see Fig. 1 (left) for an example. MacMahon [12] introduced them at the end of the 19th century as two dimensional generalisations of ordinary partitions and proved in [14] two enumeration results: He showed that the generating function of plane partitions is given by

1.1 $$\begin{aligned} \sum _{\pi } q^{|\pi |} = \prod _{i\ge 1} \frac{1}{(1-q^i)^i}, \end{aligned}$$

where the sum is over all plane partitions and $|\pi |$ is defined as the sum of the entries of $\pi$. A plane partition $\pi$ is said to be *contained in an (a, b, c)-box* if the entries of $\pi$ are at most *c* and $\pi _{i,j} \ne 0$ implies $i\le a$ and $j \le b$. The plane partition in Fig. 1 is contained in a (3, 4, 4)-box or any box of larger size. MacMahon showed that the weighted enumeration of plane partitions inside an (*a*, *b*, *c*)-box is given by

1.2 $$\begin{aligned} \sum _{\pi } q^{|\pi |} = \prod _{i=1}^a\prod _{j=1}^b\prod _{k=1}^c \frac{1-q^{i+j+k-1}}{1-q^{i+j+k-2}}, \end{aligned}$$

where the sum is over all plane partitions contained in an (*a*, *b*, *c*)-box.

While plane partitions were already introduced at the end of the 19th century, they came into the focus of the combinatorics community mainly in the second half of the last century. One major goal was to prove the enumeration formulas for the ten symmetry classes of plane partitions. These classes are defined via combinations of the three operations *reflection*, *rotation* and *complementation* which are best defined by viewing plane partitions as lozenge tilings: first, we represent a plane partition $\pi$ as stacks of unit cubes by placing $\pi _{i,j}$ unit cubes at the position (*i*, *j*), see Fig. 1 (middle). By further displaying the shape of the (*a*, *b*, *c*)-box in which we regard the plane partition in and forgetting the shading of the cubes, we obtain a lozenge tiling of a hexagon with side lengths *a*, *b*, *c*, *a*, *b*, *c*, see Fig. 1 (right). Interestingly, this was first observed by David and Tomei [7] in 1989. The operation *reflection* is defined as vertical reflection of the lozenge tiling, *rotation* as rotation by 120 degrees and *complementation* as rotation by 180 degree. While MacMahon [13] already considered plane partitions invariant under reflection, the operation complementation was first described by Mills et al. [15] in 1986. A systematic study of the 10 symmetry classes which are defined through these operations was initiated by Stanley [18, 19] and finished in 2011 by Koutschan et al. [9]. For a more detailed overview, see [11].

Already in 1916, MacMahon [14] introduced a further generalisation of partitions to arbitrary dimension, namely *higher dimensional partitions*. A *d-dimensional partition* $\pi$ is an array $(\pi _{i_1,\ldots ,i_d})$ of non-negative integers with all but finitely many entries equal to 0, such that $\pi _{{\textbf{i}}} \ge \pi _{{\textbf{i}}+e_k}$ for all indices ${\textbf{i}}=(i_1,\ldots ,i_d)$ and $1 \le k \le d$, where $e_k$ denotes the *k*-th unit vector. We say that $\pi$ is contained in an $(n_1,\ldots ,n_{d+1})$-box if all entries are at most $n_{d+1}$ and $\pi _{{\textbf{i}}}>0$ implies that $i_j \le n_j$ for all $1 \le j \le d$. Contrary to dimension 1 (partitions) and dimension 2 (plane partitions), there are hardly any results known for higher dimensional partitions in dimension 3 or higher. MacMahon conjectured a generating formula for each dimension *d* but it was disproved by Atkin et al. [2] in 1967; see also [8]. Only recently the first enumeration result for higher dimensional partitions was presented by Amanov and Yeliussizov [1]. They were able to “correct” MacMahon’s formula and showed that

1.3 $$\begin{aligned} \sum _{\pi } t^{\text {cor}(\pi )} q^{|\pi |_{\text {ch}}} = \prod _{i \ge 1} (1-t q^i)^{-\left( {\begin{array}{c}i+d-2\\ d-1\end{array}}\right) }, \end{aligned}$$

where the sum is over all *d*-dimensional partitions, and $\text {cor}$ and $|\cdot |_{\text {ch}}$ are certain statistics defined in [1, Sects. 4 and 5].

In this paper we introduce a new symmetry class for plane partitions, namely *quarter complementary plane partitions* (QCPPs), which can be generalised immediately to higher dimensional partitions. Instead of presenting the definition for QCPPs (it follows from the corresponding definition for higher dimensional partitions in Sect. 2.2) we aim to convey the geometric intuition of this symmetry class next.

**Fig. 2:**
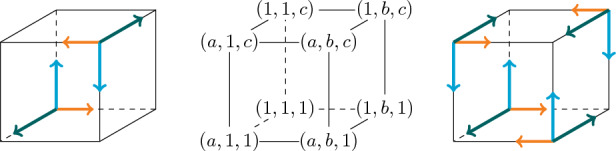
The labels of the corners of an (*a*, *b*, *c*)-box (middle), the corners where a copy of $\pi$ is placed for self-complementary plane partitions (left), and the corners where copies of $\pi$ are placed for quarter complementary plane partitions (right). The colour and lengths of the arrows indicate the orientation of the copies

Let $\pi =(\pi _{i,j})$ be a plane partition inside an (*a*, *b*, *c*)-box and define by $\pi ^\prime =(\pi _{a+1-i,b+1-j})_{i,j}$ the “dual plane partition” of $\pi$ inside the (*a*, *b*, *c*)-box. Geometrically, we think of the dual plane partition as stacks of unit cubes hanging from the ceiling of the (*a*, *b*, *c*)-box instead of standing at its floor, where the first stack is positioned at the corner with coordinates (*a*, *b*, *c*) instead of the corner with coordinates (1, 1, 1), see Fig. 2 (left) for a sketch. It is not difficult to see, compare for example with [11, Sect. 6], that $\pi$ is *self-complementary* if $\pi$ and $\pi ^\prime$ fill the (*a*, *b*, *c*)-box without overlap when regarded as stacks of unit cubes. In the array perspective this means $\pi _{i,j}+\pi _{i,j}^\prime = c$ for all $1 \le i \le a$ and $1 \le j \le b$. We can now generalise this idea. Let *C* be a set of corners of the (*a*, *b*, *c*)-box. We define a plane partition $\pi$ to be *C-complementary* if we can fill the (*a*, *b*, *c*)-box without overlap by copies of $\pi$ placed at the corners of *C* similar to before, i.e. if a copy of $\pi$ is placed at a corner of the form $(*,*,c)$ then its stacks of unit cubes hang from the ceiling of the box instead of standing on its floor. It is immediate that such a $\pi$ can only exist if $|C|\in \{1,2,4,8\}$. We can ignore the cases $|C|=1$ and $|C|=8$ since they are trivial.

For $|C|=2$, there are up to rotation three possible configurations $C_1=\{(1,1,1),(a,1,1)\}$, $C_2=\{(1,1,1),(a,b,1)\}$, and $C_3=\{(1,1,1),(a,b,c)\}$. It is immediate that there is only one $C_1$-complementary plane partition if *a* is even and none otherwise. Each $C_2$-complementary plane partition is uniquely determined by its boxes of the form (*x*, *y*, 1). Hence, we can map them bijectively to the set of partitions $\lambda =(\lambda _1,\ldots ,\lambda _a)$ inside an (*a*, *b*)-box which satisfy $\lambda _i+\lambda _{a+1-i}=b$. As described before, $C_3$-complementary plane partitions are exactly self-complementary plane partitions.

For $|C|=4$, there are up to rotation six possible configurations:

$$\begin{aligned} C_4&= \{ (1,1,1),(a,1,1),(1,b,1),(a,b,1)\},\\ C_5&= \{ (1,1,1),(a,1,1),(1,b,1),(1,1,c)\},\\ C_6&= \{ (1,1,1),(a,1,1),(1,b,1),(a,1,c)\},\\ C_7&= \{ (1,1,1),(a,1,1),(1,b,1),(a,b,c)\},\\ C_8&= \{ (1,1,1),(a,b,1),(1,1,c),(a,b,c)\},\\ C_9&= \{ (1,1,1),(a,b,1),(1,b,c),(a,1,c)\}, \end{aligned}$$

see Fig. 2 (right) for a sketch of $C_9$. There is only one $C_4$-complementary plane partition if 2 divides both *a* and *b* and none otherwise, analogously for $C_7$. It is immediate that there are no $C_5$- or $C_6$-complementary plane partitions and that $C_8$-complementary plane partitions do not exist for odd *c* and are in bijection to $C_2$-complementary plane partitions otherwise. Finally, $C_9$-complementary plane partitions are the objects we want to call *quarter complementary plane partitions*.

In Sect. 2, we generalise this geometric approach to higher dimensions using *higher dimensional Ferrers diagrams*. Translating the obtained criteria on Ferrers diagrams back to the array description for higher dimensional partitions, we obtain in Lemma 2.2 the definition of *d-dimensional fully complementary partitions* (FCPs). In Proposition 2.4, we describe the recursive structure of FCPs which implies immediately our main result.

### Theorem 1.1

Let ${\textbf{x}}=(x_1,\ldots ,x_{d+1})$, ${\textbf{n}}=(n_1,\ldots ,n_{d+1}) \in {\mathbb {N}}_{>0}^{d+1}$ and denote by ${{\,\textrm{FCP}\,}}({\textbf{n}})$ the set of fully complementary partitions inside a $(2n_1,\ldots ,2n_{d+1})$-box. Then,

1.4 $$\begin{aligned} \sum _{{\textbf{n}} \in {\mathbb {N}}^{d+1}_{>0}} |{{\,\textrm{FCP}\,}}({\textbf{n}})| {\textbf{x}}^{{\textbf{n}}} = \prod \limits _{i=1}^{d+1}\frac{x_i}{1-x_i} \cdot \frac{\sum _{i=1}^{d+1}(1- x_i)}{1-\sum _{i=1}^{d+1} x_i}. \end{aligned}$$

The recursive structure of FCPs can be further used to construct a bijection between *d*-dimensional FCPs and lattice paths in the positive $d+1$-dimensional orthant starting from any integer point in the interior of its *d*-dimensional boundary.

In Sect. 3, we consider the “typical” symmetry classes for plane partitions restricted to two-dimensional FCPs, i.e. quarter complementary plane partitions. It turns out that there exists at most one symmetric QCPP in an (*a*, *b*, *c*)-box and that a QCPP can be neither cyclically symmetric nor transpose-complementary. By introducing two variations, namely *quasi-symmetric* and *quasi-transpose-complementary*, we are able to show enumerative results for the corresponding symmetry classes and combinations thereof for QCPPs, see Proposition 3.1 to Proposition 3.4. It is an immediate question if the enumeration of plane partitions under these variations of symmetry classes have closed expressions. In Sect. 4, we consider these new symmetry classes and similar generalisations. We present three conjectures, the corresponding data generated in computer experiments are given in Appendix A, and the proof of the next result.

### Theorem 1.2

A plane partition $\pi$ inside an (*n*, *n*, *c*)-box is called *quasi-transpose-complementary* if $\pi _{i,j}+\pi _{n+1-j,n+1-i}=c$ holds for all $1 \le i,j \le n$ with $i \ne n+1-j$. The number of quasi-transpose-complementary plane partitions inside an (*n*, *n*, *c*)-box is equal to the number of symmetric plane partitions inside an (*n*, *n*, *c*)-box.

We present different proofs of the above theorem and discuss how it is related to known results on plane partitions, lozenge tilings and perfect matchings, respectively. For odd *c*, we relate quasi-transpose-complementary plane partitions to transpose-complementary plane partitions and obtain immediately the following numerical connection between symmetric and transpose-complementary plane partitions which seems to be new

1.5 $$\begin{aligned} 2^{n-1} {{\,\textrm{TCPP}\,}}(n,n,2c)= {{\,\textrm{SPP}\,}}(n-1,n-1,2c+1), \end{aligned}$$

where ${{\,\textrm{TCPP}\,}}(n,n,2c)$ denotes the number of transpose-complementary plane partitions inside an (*n*, *n*, 2*c*)-box and ${{\,\textrm{SPP}\,}}(n,n,c)$ denotes the number of symmetric plane partitions inside an (*n*, *n*, *c*)-box. Since the proof of the above theorem is computational, it is still an open question if one can find a bijection between symmetric plane partitions and quasi-transpose-complementary plane partitions.

## Fully Complementary Partitions

### Fully Complementary Ferrers Diagrams

A *d-dimensional Ferrers diagram* $\lambda$ is a finite subset of $({\mathbb {N}}_{>0})^{d+1}$ such that $(x_1,\ldots ,x_{d+1}) \in \lambda$ implies $(y_1,\ldots ,y_{d+1}) \in \lambda$ whenever $1 \le y_i \le x_i$ for all $1 \le i \le d+1$. Equivalently, $\lambda$ is an order ideal in the poset $({\mathbb {N}}_{>0})^{d+1}$ where the order relation is component-wise the order relation of the integers. For a positive integer *n*, we define $[n]=\{1,\ldots ,n\}$. We say that $\lambda$ is *contained* in an $(n_1,\ldots ,n_{d+1})$-box for positive integers $n_1,\ldots , n_{d+1}$, if $\lambda$ is a subset of $[n_1] \times \cdots \times [n_{d+1}]$. It is immediate that the map

$$\begin{aligned} \lambda \mapsto \pi (\lambda ) = \big ( |\{ (i_1,\ldots ,i_d,k)\in \lambda \}| \big )_{i_1,\ldots ,i_d} \end{aligned}$$

is a bijection between *d*-dimensional Ferrers diagrams and *d*-dimensional partitions which respects the property of being contained in an $(n_1,\ldots ,n_{d+1})$-box. Therefore, we identify for the remainder of this paper a *d*-dimensional Ferrers diagram with the *d*-dimensional partition it is mapped to.

Let ${\textbf{n}}=(n_1,\ldots ,n_{d+1})$ be a sequence of positive integers and $I \subseteq [d+1]$. We define the bijection $\rho _{I,{\textbf{n}}}$ from $[2n_1] \times \cdots \times [2n_{d+1}]$ onto itself as

$$\begin{aligned} \rho _{I,{\textbf{n}}}(x_1,\ldots ,x_{d+1}):= \left( {\left\{ \begin{array}{ll} x_i \qquad &{} i \notin I,\\ 2n_i+1-x_i &{} i \in I, \end{array}\right. } \right) _{1 \le i \le d+1}. \end{aligned}$$

If ${\textbf{n}}$ is clear from the context, we will omit it and write $\rho _I$ instead of $\rho _{I,{\textbf{n}}}$. We can use the map $\rho _I$ to rephrase the definition of *quarter complementary plane partitions*. Let $\lambda$ be a two-dimensional Ferrers diagram contained in a $(2n_1,2n_2,2n_3)$-box. Then, $\rho _{\{1,2\}}(\lambda )$ corresponds to the copy of $\lambda$ placed in the corner $(2n_1,2n_2,1)$ of the box, $\rho _{\{1,3\}}(\lambda )$ to the copy of $\lambda$ placed in the corner $(2n_1,1,2n_3)$ and $\rho _{\{2,3\}}(\lambda )$ to the copy placed in the corner $(1,2n_2,2n_3)$. Hence, $\lambda$ corresponds to a QCPP if $\lambda , \rho _{\{1,2\}}(\lambda ), \rho _{\{1,3\}}(\lambda ), \rho _{\{2,3\}}(\lambda )$ have pairwise empty intersection and their union is equal to $[2n_1]\times [2n_2] \times [2n_3]$. We extend this definition to any dimension *d*.

#### Definition 2.1

Let $n_1,\ldots ,n_{d+1}$ be positive integers. A *d*-dimensional Ferrers diagram $\lambda$ is called *fully complementary* inside a $(2n_1,\ldots ,2n_{d+1})$-box if for all pairs of subsets $I,J \subseteq [d+1]$ of even size $\rho _I(\lambda ) \cap \rho _J(\lambda ) = \emptyset$ holds and

$$\begin{aligned} \bigcup _{\begin{array}{c} I \subseteq [d+1]\\ |I| \text { even} \end{array}}\rho _I(\lambda )= [2n_1] \times \cdots \times [2n_{d+1}]. \end{aligned}$$

It is easy to see that $\rho _I(\lambda ) \cap \rho _J(\lambda ) = \emptyset$ holds for all subsets $I,J \subseteq [d+1]$ of even size exactly if $\lambda \cap \rho _I(\lambda ) = \emptyset$ holds for all subsets $I \subseteq [d+1]$ of even size.

The three-dimensional Ferrers diagram $\lambda =\{(1,1,1,1),(2,1,1,1)\}$ is fully complementary inside a (2, 2, 2, 2)-box. This can be seen easily by calculating the according images under $\rho _I$ for even sized *I* which are given by

$$\begin{aligned}&\rho _{\{1,2\}}(\lambda )= \{(2,2,1,1),(1,2,1,1)\},&\rho _{\{2,3\}}(\lambda )= \{(1,2,2,1),(2,2,2,1)\}, \\&\rho _{\{1,3\}}(\lambda )= \{(2,1,2,1),(1,1,2,1)\},&\rho _{\{2,4\}}(\lambda )= \{(1,2,1,2),(2,2,1,2)\}, \\&\rho _{\{1,4\}}(\lambda )= \{(2,1,1,2),(1,1,1,2)\},&\rho _{\{3,4\}}(\lambda )= \{(1,1,2,2),(2,1,2,2)\}, \\&\rho _{\{1,2,3,4\}}(\lambda )= \{(2,2,2,2),(1,2,2,2)\}. \end{aligned}$$

There are three further Ferrers diagrams which are fully complementary inside (2, 2, 2, 2), namely $\{(1,1,1,1),(1,2,1,1)\}$, $\{(1,1,1,1),(1,1,2,1)\}$ and $\{(1,1,1,1),(1,1,1,2)\}$. More generally, define the *d*-dimensional Ferrers diagrams

2.1 $$\begin{aligned} \lambda ^{d,i}:=\{(1,\ldots ,1),(1,\ldots ,1,2,1,\ldots ,1)\}, \end{aligned}$$

where the 2 entry is at position *i*. Then, the *d*-dimensional Ferrers diagrams which are fully complementary inside the box $(2,\ldots ,2)$ are exactly $\lambda ^{d,1},\ldots ,\lambda ^{d,d+1}$.

In the above definition of fully complementary, we restricted ourselves to boxes with even side lengths. The definition can be extended to any side lengths, however, as we see next, either there are no such Ferrers diagrams, or we obtain a set of Ferrers diagram which is in bijection to one where the box has only even side lengths. First, assume that there exist $k<l$ such that the *k*-th and *l*-th side lengths of the box are given by $2n_k+1$ and $2n_l+1$, respectively, and that $\lambda$ is a Ferrers diagram which is fully complementary inside this box. Let us regard the point $P=(1,\ldots ,1,n_k,1,\ldots ,1,n_l,1,\ldots ,1)$ whose components are 1 except for the *k*-th and *l*-th component. Then, there exists an $I\subset [d+1]$ of even size such that $P \in \rho _{I,{\widehat{{\textbf{n}}}}}(\lambda )$, where ${\widehat{{\textbf{n}}}}=({\widehat{n}}_1,\ldots ,{\widehat{n}}_{d+1})$ and ${\widehat{n}}_i$ is half of the side length of the box in the direction of *i*-th standard vector. Let $I^\prime$ be the set $I^\prime = \left( I {\setminus } \{k,l \} \right) \cup \left( \{k,l\} {\setminus } I \right)$. It is immediate that $|I^\prime |$ is even and that $P \in \rho _{I^\prime ,{\widehat{{\textbf{n}}}}}(\lambda )$ which is a contradiction. Hence, there exists no fully complementary Ferrers diagram inside a box with at least two odd side lengths.

Now, let all side lengths of the box be even with the exception of one side length. Without loss of generality, we can assume that the box is a $(2n_1,\ldots ,2n_d,2n_{d+1}+1)$-box. For a fully complementary partition $\pi$ in this box, we see that a point of the form $(i_1,\ldots ,i_d,n_{d+1}+1)$ is exactly in $\pi$ if $i_j \le n_j$ for all $1 \le j \le d$. The map

$$\begin{aligned} \Psi : \lambda\mapsto & {} \{ (i_1,\ldots ,i_d,i_{d+1}) \in \lambda : i_{d+1} \le n_{d+1} \} \\{} & {} \cup \{ (i_1,\ldots ,i_d,i_{d+1}-1) \in \lambda : i_{d+1} > n_{d+1}+1 \}, \end{aligned}$$

is a surjection from Ferrers diagrams inside a $(2n_1,\ldots ,2n_d,2n_{d+1}+1)$-box to Ferrers diagrams inside a $(2n_1,\ldots ,2n_d,2n_{d+1})$-box. It is not difficult to see that the map $\Psi$ commutes with $\rho _I$ for each $I \subseteq [d+1]$, i.e.

$$\begin{aligned} \Psi \circ \rho _{I,(n_1,\ldots ,n_d,n_{d+1}+\frac{1}{2})} = \rho _{I,(n_1,\ldots ,n_{d+1})} \circ \Psi . \end{aligned}$$

Hence, $\Psi$ maps fully complementary Ferrers diagrams inside a $(2n_1,\ldots ,2n_d,2n_{d+1}+1)$-box bijectively to those inside a $(2n_1,\ldots ,2n_{d+1})$-box.

### The Generating Function of FCPs

We call a *d*-dimensional partition $\pi$ *fully complementary* inside a $(2n_1,\ldots ,2n_{d+1})$-box if its corresponding Ferrers diagram is fully complementary in this box. The fully complementary partitions inside the (2, 2, 2, 2)-box are shown next where we write $(\pi _{i,j,1})$ in the top row and $(\pi _{i,j,2})$ in the row below:

$$\begin{aligned} \begin{matrix} 1 &{} 1 \\ 0 &{} 0 \end{matrix} \qquad \qquad \qquad \begin{matrix} 1 &{} 0 \\ 1 &{} 0 \end{matrix} \qquad \qquad \qquad \begin{matrix} 1 &{} 0 \\ 0 &{} 0 \end{matrix} \qquad \qquad \qquad \begin{matrix} 2 &{} 0 \\ 0 &{} 0 \end{matrix}\\ \\ \begin{matrix} 0 &{} 0 \\ 0 &{} 0 \end{matrix} \qquad \qquad \qquad \begin{matrix} 0 &{} 0 \\ 0 &{} 0 \end{matrix} \qquad \qquad \qquad \begin{matrix} 1 &{} 0 \\ 0 &{} 0 \end{matrix} \qquad \qquad \qquad \begin{matrix} 0 &{} 0 \\ 0 &{} 0 \end{matrix} \end{aligned}$$

For ${\textbf{n}}=(n_1,\ldots ,n_{d+1})$ and a subset $I \subseteq [d]$, we define the map $\gamma _{I,{\textbf{n}}}$ as

$$\begin{aligned} \gamma _{I,{\textbf{n}}}(\pi ) = \left( \pi _{\rho _{I,{\textbf{n}}}(i_1,\ldots ,i_n)} \right) _{i_1,\ldots ,i_n}. \end{aligned}$$

Again we omit the subscript ${\textbf{n}}$ and write $\gamma _I$ whenever ${\textbf{n}}$ is clear from context. The next lemma rephrases the conditions of being fully complementary directly for a *d*-dimensional partition.

#### Lemma 2.2

Let $n_1,\ldots , n_{d+1}$ be positive integers. A *d*-dimensional partition $\pi$ is fully complementary inside a $(2n_1,\ldots ,2n_{d+1})$-box if and only if

2.2 $$\begin{aligned} \pi _{i_1,\ldots ,i_d} \cdot \gamma _J(\pi )_{i_1,\ldots ,i_d}=0, \end{aligned}$$

2.3 $$\begin{aligned} \sum _{I \subseteq [d]}\gamma _I(\pi )_{i_1,\ldots ,i_d} = 2n_{d+1}, \end{aligned}$$

for all non-empty subsets $J \subseteq [d]$ of even size and for all $(i_1,\ldots ,i_d) \in [2n_1] \times \cdots \times [2n_d]$.

#### Proof

Let $\lambda$ be a fully complementary Ferrers diagram inside a $(2n_1,\ldots ,2n_{d+1})$-box and $(\pi _{i_1,\ldots ,i_d})_{i_1,\ldots ,i_d}$ the corresponding *d*-dimensional partition. For $I \subseteq [d]$, we have

$$\begin{aligned} \gamma _I(\pi )_{i_1,\ldots ,i_d}= {\left\{ \begin{array}{ll} |\{(i_1,\ldots ,i_d,k) \in \rho _I (\lambda ): k \in {\mathbb {N}}_{>0}\}| \qquad \qquad &{} |I| \text { is even}, \\ |\{(i_1,\ldots ,i_d,k) \in \rho _{I\cup \{d+1\}} (\lambda ): k \in {\mathbb {N}}_{>0}\}| &{} |I| \text { is odd}. \end{array}\right. } \end{aligned}$$

By definition, the intersection $\lambda \cap \rho _{I}(\lambda )$ is empty for even sized *I* exactly if $\pi _{i_1,\ldots ,i_d} \cdot \gamma _I(\pi )_{i_1,\ldots ,i_d}=0$ for all $(i_1,\ldots ,i_d) \in [2n_1] \times \cdots \times [2n_d]$. For odd sized $I \subseteq [d]$, the intersection $\lambda \cap \rho _{I \cup \{d+1\}}(\lambda )$ is empty if and only if $\pi _{i_1,\ldots ,i_d}+\gamma _I(\pi )_{i_1,\ldots ,i_d} \le 2n_{d+1}$ for all $(i_1,\ldots ,i_d) \in [2n_1] \times \cdots \times [2n_d]$. Finally, the union of all $\rho _J(\lambda )$ with $J \subseteq [d+1]$ of even size is exactly $[2n_1]\times \cdots \times [2n_{d+1}]$ if and only if (2.3) is satisfied for all $(i_1,\ldots ,i_d) \in [2n_1] \times \cdots \times [2n_d]$. $\square$

#### Remark 2.3

Let $\pi \in {{\,\textrm{FCP}\,}}({\textbf{n}})$ and let $i_1,\ldots ,i_d$ such that $0<\pi _{i_1,\ldots ,i_d} <2n_{d+1}$. The above Lemma implies that there exists exactly one $I \subseteq [d]$ of odd size such that $\pi _{i_1,\ldots ,i_d} + \gamma _I(\pi )_{i_1,\ldots ,i_d}=2n_{d+1}$. By (2.3), there exists at least one set $I_1$ of odd size such that $\gamma _{I_1}(\pi )_{i_1,\ldots ,i_d} >0$. By definition, this implies $(i_1,\ldots ,i_d,1) \in \rho _{I_1\cup \{d+1\}}(\lambda )$ where $\lambda$ is the corresponding Ferrers diagram of $\pi$. Hence, by Definition 2.1, there cannot exist another $I_2$ with the same properties.

Denote by ${{\,\textrm{FCP}\,}}(n_1,\ldots ,n_{d+1})$ the set of fully complementary partitions inside a $(2n_1,\ldots ,2n_{d+1})$-box. For $1 \le k \le d$, we define the map^1^$\varphi _k: {{\,\textrm{FCP}\,}}(n_1,\ldots ,n_{d+1}) \rightarrow {{\,\textrm{FCP}\,}}(n_1,\ldots ,n_k+1,\ldots ,n_{d+1})$ as

$$\begin{aligned} \varphi _k(\pi )_{i_1,\ldots ,i_d}= {\left\{ \begin{array}{ll} \pi _{i_1,\ldots ,i_d} \qquad &{} i_k \le n_k, \\ n_{d+1} &{} i_k \in \{n_k+1,n_k+2\} \text { and }\\ &{}\qquad i_j \le n_j \text { for all } 1 \le j \ne k \le d,\\ \pi _{i_1,\ldots ,i_k-2,\ldots ,i_d} &{} i_k>n_k+2,\\ 0 &{} \text {otherwise}, \end{array}\right. } \end{aligned}$$

and the map $\varphi _{d+1}: {{\,\textrm{FCP}\,}}(n_1,\ldots ,n_{d+1}) \rightarrow {{\,\textrm{FCP}\,}}(n_1,\ldots ,n_d,n_{d+1}+1)$ as

$$\begin{aligned} \varphi _{d+1}(\pi )_{i_1,\ldots ,i_d}= {\left\{ \begin{array}{ll} \pi _{i_1,\ldots ,i_d}+2 \qquad &{} i_j \le n_j \text { for all }1 \le j \le d,\\ \pi _{i_1,\ldots ,i_d} &{} \text { otherwise}. \end{array}\right. } \end{aligned}$$

It is not difficult to see that these maps are well defined. Let $\pi$ be the fully complementary partition inside the (4, 4, 4)-box displayed next:

$$\begin{aligned} \begin{array}{cccc} 4 &{} 2 &{} 2 &{} 0 \\ 3 &{} 2 &{} 2 &{} 0 \\ 1 &{} 0 &{} 0 &{} 0 \\ 0 &{} 0 &{} 0 &{} 0 \end{array} \end{aligned}$$

The images of $\pi$ under the maps $\varphi _1,\varphi _2$ or $\varphi _3$, respectively, are given as follows:

$$\begin{aligned} \begin{array}{cccc} 4 &{} 2 &{} 2 &{} 0 \\ 3 &{} 2 &{} 2 &{} 0 \\ 2 &{} 2 &{} 0 &{} 0 \\ 2 &{} 2 &{} 0 &{} 0 \\ 1 &{} 0 &{} 0 &{} 0 \\ 0 &{} 0 &{} 0 &{} 0 \end{array} \qquad \qquad \begin{array}{cccccc} 4 &{} 2 &{} 2 &{} 2 &{} 2 &{} 0 \\ 3 &{} 2 &{} 2 &{} 2 &{} 2 &{} 0 \\ 1 &{} 0 &{} 0 &{} 0 &{} 0 &{} 0 \\ 0 &{} 0 &{} 0 &{} 0 &{} 0 &{} 0 \end{array} \qquad \qquad \begin{array}{cccc} 6 &{} 4 &{} 2 &{} 0 \\ 5 &{} 4 &{} 2 &{} 0 \\ 1 &{} 0 &{} 0 &{} 0 \\ 0 &{} 0 &{} 0 &{} 0 \end{array} \end{aligned}$$

As we see in a moment, it is useful to extend the definition of fully complementary partitions to “empty boxes”. In particular, we define ${{\,\textrm{FCP}\,}}(n_1,\ldots ,n_{d+1})$ to consist of the “empty array” in case that one $n_{k_0}$ is equal to 0 and all other $n_i$ are positive. We extend the map $\varphi _k$ to these sets, where $\varphi _k$ is the identity (mapping the empty array onto the empty array) if $k\ne k_0$ and mapping the empty array to

$$\begin{aligned} \left( {\left\{ \begin{array}{ll} n_{d+1} \qquad &{}i_j \le n_j \text { for all } 1 \le j \ne k_0 \le d,\\ 0 &{} \text { otherwise,} \end{array}\right. } \right) _{i_1,\ldots ,i_d}, \end{aligned}$$

if $k=k_0 \ne d+1$ and to

$$\begin{aligned} \left( {\left\{ \begin{array}{ll} 2 \qquad &{}i_j \le n_j \text { for all } 1 \le j \le d,\\ 0 &{} \text { otherwise,} \end{array}\right. } \right) _{i_1,\ldots ,i_d}, \end{aligned}$$

if $k=k_0=d+1$. The maps $\varphi _k$ allow us to prove the following recursive structure for FCPs.

#### Proposition 2.4

Let ${\textbf{n}}=(n_1,\ldots ,n_{d+1})$ be a sequence of positive integers. Then, ${{\,\textrm{FCP}\,}}({\textbf{n}})$ is equal to the disjoint union

2.4 $$\begin{aligned} {{\,\textrm{FCP}\,}}({\textbf{n}}) = {\dot{\bigcup }}_{1 \le k \le d+1} \varphi _k\big ({{\,\textrm{FCP}\,}}({\textbf{n}}-e_k) \big ), \end{aligned}$$

where $e_k$ is the *k*-th unit vector and ${\dot{\bigcup }}$ denotes the disjoint union.

#### Proof

First, we prove that the images of the $\varphi _k$ are disjoint. Let $k<l$ be integers with $\varphi _k\big ({{\,\textrm{FCP}\,}}({\textbf{n}}-e_k)\big ) \cap \varphi _l\big ({{\,\textrm{FCP}\,}}({\textbf{n}}-e_l)\big ) \ne \emptyset$ and let $\pi$ be an element of this intersection. First, we assume that $l=d+1$. Since $\pi \in \varphi _k({\textbf{n}}-e_k)$, we have by definition $\pi _{n_1,\ldots ,n_d}=n_{d+1}$. On the other hand, $\pi \in \varphi _{d+1}\big ({{\,\textrm{FCP}\,}}({\textbf{n}}-e_{d+1})\big )$ implies $\pi _{n_1,\ldots ,n_{d}} \ge n_{d+1}+1$ which is a contradiction. Now let $l < d+1$. By definition of $\varphi _k,\varphi _l$, we have

$$\begin{aligned} \pi _{{\textbf{n}}+e_k} = \pi _{{\textbf{n}}+e_l} =n_{d+1}. \end{aligned}$$

This implies

$$\begin{aligned} \pi _{{\textbf{n}}+e_k} \cdot \left( \gamma _{\{k,l\}} (\pi ) \right) _{{\textbf{n}}+e_k} =\pi _{{\textbf{n}}+e_k} \cdot \pi _{{\textbf{n}}+e_l} = n_{d+1}^2 \ne 0, \end{aligned}$$

which contradicts (2.2). Hence, the union in (2.4) is disjoint.

$$\square $$

Let $\pi \in {{\,\textrm{FCP}\,}}({\textbf{n}})$. It is easy to see that $\pi _{{\textbf{n}}} \ge n_{d+1}$. If $\pi _{{\textbf{n}}} \ge n_{d+1}+1$, then $\pi \in \varphi _{d+1}\big ({{\,\textrm{FCP}\,}}({\textbf{n}}-e_{d+1})\big )$. Hence, let us assume that $\pi _{{\textbf{n}}}=n_{d+1}$. By (2.2), $\gamma _I(\pi )_{{\textbf{n}}}=0$ for all $I \subset [d]$ of even size. Remark 2.3 implies that there exists exactly one $I \subseteq [d]$ of odd size with $\pi _{{\textbf{n}}}+\gamma _I(\pi )_{{\textbf{n}}}=2n_{d+1}$ which implies $\gamma _I(\pi )_{{\textbf{n}}} = \pi _{\rho _I({\textbf{n}})}=n_{d+1}$. Let $k \in I$ and define ${\widehat{{\textbf{n}}}}=(1,\ldots ,n_{k},\ldots ,1)$ as the vector whose entries are 1 except on position *k*, where the entry is $n_{k}$ and ${\widehat{{\textbf{i}}}}=(i_1,\ldots ,i_d)$ as the vector with $i_k=n_k$ and $i_j \le n_j$ for all $1 \le j \ne k \le d$. Since $\pi$ is a *d*-dimensional partition, we have the following inequalities: 
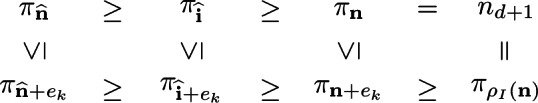
 Since $\pi _{\rho _{\{k\}}({\textbf{n}})}=\pi _{{\textbf{n}}+e_k}>0$ and there exists only one non-empty *I* with $\pi _{\rho _I({\textbf{n}})} \ne 0$, this implies that $I=\{k\}$ and hence $\pi _{{\textbf{n}}+e_k}=n_{d+1}$. Furthermore, we have $\pi _{{\widehat{{\textbf{n}}}}} +\pi _{{\widehat{{\textbf{n}}}}+e_{k}}\ge \pi _{{\textbf{n}}}+\pi _{{\textbf{n}}+e_k} =2n_{d+1}$ by the above inequalities and $\pi _{{\widehat{{\textbf{n}}}}} +\pi _{{\widehat{{\textbf{n}}}}+e_{k}}\le 2n_{d+1}$ by (2.3) since $\gamma _{\{k\}}(\pi )_{{\widehat{{\textbf{n}}}}}=\pi _{{\widehat{{\textbf{n}}}}+e_{k}}$. This implies $\pi _{{\widehat{{\textbf{n}}}}}= \pi _{{\widehat{{\textbf{n}}}}+e_{k}}=n_{d+1}$ and hence $\pi _{{\widehat{i}}}=\pi _{{\widehat{i}}+e_k}=n_{d+1}$ for all ${\widehat{i}}$ defined as before. Denote for $l \ne k$ by ${\widehat{{\textbf{n}}}}_l$ the vector with all components equal to 1 except of the *k*-th and *l*-th component which are $n_k$ or $n_l$, respectively. Since $\rho _{\{k,l\}}({\widehat{{\textbf{n}}}}_l) = {\widehat{{\textbf{n}}}}_l+e_k+e_l$ and $\rho _{\{k,l\}}({\widehat{{\textbf{n}}}}_l+e_k) = {\widehat{{\textbf{n}}}}_l+e_l$ it follows from (2.2) that $\pi _{{\widehat{{\textbf{n}}}}+e_l} =\pi _{{\widehat{{\textbf{n}}}}+e_l+e_k} =0$ and hence $\pi _{i_1,\ldots ,i_d}=0$ for all $(i_1,\ldots ,i_d)$ such that $i_k\in \{n_k,n_k+1\}$ and there exists an $i_j >n_j$. Denote by $\pi ^\prime$ the array obtained by deleting all entries for which the *k*-th component of the index is either $n_k$ or $n_k+1$. It is not difficult to verify that $\pi ^\prime \in {{\,\textrm{FCP}\,}}({\textbf{n}}-e_{k})$ and $\pi = \varphi _{k}(\pi ^\prime )$ which proves the claim. $\square$

#### Remark 2.5

Let $\pi \in {{\,\textrm{FCP}\,}}({\textbf{n}})$. As a consequence of the above theorem, we can find the *k* such that $\pi \in \varphi _k({{\,\textrm{FCP}\,}}({\textbf{n}}-e_k))$ easily as follows. First, restrict $\pi$ to its central hypercube *B* of size $(2,\ldots ,2)$. It is easy to see that this again fully complementary inside *B*. As stated in the paragraph below (2.1), the restriction is equal to some $\lambda ^{d,i}$. By comparing with the definition of $\varphi _k$, we obtain that it has to be $\lambda ^{d,k}$.

As we see next, Theorem 1.1 is now a direct consequence of the above proposition.

#### Proof of Theorem 1.1

Let us denote by $Z({\textbf{x}})$ the left hand side of (1.4), i.e. $Z({\textbf{x}}) = \sum _{{\textbf{n}}\in {\mathbb {N}}^{d+1}_{>0}} |{{\,\textrm{FCP}\,}}({\textbf{n}})|{\textbf{x}}^{{\textbf{n}}}$. Denote further by $Z_j({\textbf{x}})= \sum _{{\textbf{n}}}|{{\,\textrm{FCP}\,}}({\textbf{n}})|{\textbf{x}}^{{\textbf{n}}}$, where the sum is over all ${\textbf{n}}\in {\mathbb {N}}^{d+1}$ where the *j*-th component is 0 and all the other components are positive. It is immediate that

$$\begin{aligned} Z_j({\textbf{x}}) = \prod _{\begin{array}{c} 1 \le i \le d+1 \\ i\ne j \end{array}}\frac{x_i}{1-x_i}, \end{aligned}$$

since each of the ${{\,\textrm{FCP}\,}}({\textbf{n}})$ in the sum has exactly one element, namely the “empty array”. Using Proposition 2.4, we rewrite $Z({\textbf{x}})$ as

$$\begin{aligned} Z({\textbf{x}})= & {} \sum _{{\textbf{n}}\in {\mathbb {N}}_{>0}^{d+1}}\left| {\dot{\bigcup }}_{i=1}^{d+1} \varphi _i({{\,\textrm{FCP}\,}}({\textbf{n}}-e_i))\right| {\textbf{x}}^{{\textbf{n}}} = \sum _{i=1}^{d+1}x_i \sum _{{\textbf{n}}\in {\mathbb {N}}_{>0}^{d+1}} \left| {{\,\textrm{FCP}\,}}({\textbf{n}}-e_i)\right| {\textbf{x}}^{{\textbf{n}}-e_i} \\= & {} \sum _{i=1}^{d+1} x_i \left( Z({\textbf{x}}) + Z_i({\textbf{x}}) \right) = Z({\textbf{x}}) \sum _{i=1}^{d+1} x_i + \prod \limits _{i=1}^{d+1}\frac{x_i}{1-x_i} \left( \sum _{i=1}^{d+1}(1- x_i)\right) . \end{aligned}$$

By bringing all $Z({\textbf{x}})$ terms on one side, we obtain the assertion. $\square$

#### Remark 2.6

Let ${\textbf{n}}=(n_1,\ldots ,n_{d+1}) \in {\mathbb {N}}_{>0}^{d+1}$ be given. By applying Proposition 2.4 iteratively, we see that each $\pi \in {{\,\textrm{FCP}\,}}({\textbf{n}})$ can be uniquely written as $\varphi _{i_k} \circ \cdots \circ \varphi _{i_1} (\sigma )$ where $\sigma$ is an “empty array” inside an appropriate “empty box” with dimension ${\textbf{n}}^\prime =(n_1^\prime ,\ldots ,n_{d+1}^\prime )$ such that $\varphi _{i_1}(\sigma )$ is a non-empty array. We map $\pi$ to the lattice path starting at ${\textbf{n}}^\prime$ and ending at ${\textbf{n}}$ whose *j*th step is $e_{i_j}$. This yields a bijection between FCPs inside a $(2n_1,\ldots ,2n_{d+1})$-box and lattice paths inside the positive $(d+1)$-dimensional orthant starting from any integer point on its *d*-dimensional boundary and ending at ${\textbf{n}}$ with step set $\{e_1,\ldots ,e_{d+1}\}$ such that all coordinates are positive after the first step. Below we show the construction for an FCP inside a (6, 4, 4)-box which is mapped to the lattice path from (1, 1, 0) to (3, 2, 2) with steps $(e_3,e_1,e_3,e_2,e_1)$:

$$\begin{aligned} \begin{array}{cccc} 4 &{} 2 &{} 2 &{} 0 \\ 3 &{} 2 &{} 2 &{} 0 \\ 2 &{} 2 &{} 0 &{} 0 \\ 2 &{} 2 &{} 0 &{} 0 \\ 1 &{} 0 &{} 0 &{} 0 \\ 0 &{} 0 &{} 0 &{} 0 \end{array} \hspace{5pt} \xleftarrow {\varphi _1} \hspace{5pt} \begin{array}{cccc} 4 &{} 2 &{} 2 &{} 0 \\ 3 &{} 2 &{} 2 &{} 0 \\ 1 &{} 0 &{} 0 &{} 0 \\ 0 &{} 0 &{} 0 &{} 0 \end{array} \hspace{5pt} \xleftarrow {\varphi _2} \hspace{5pt} \begin{array}{cc} 4 &{} 0 \\ 3 &{} 0 \\ 1 &{} 0 \\ 0 &{} 0 \end{array} \hspace{5pt} \xleftarrow {\varphi _3} \hspace{5pt} \begin{array}{cc} 2 &{} 0 \\ 1 &{} 0 \\ 1 &{} 0 \\ 0 &{} 0 \end{array} \hspace{6pt} \xleftarrow {\varphi _1} \hspace{6pt} \begin{array}{cc} 2 &{} 0 \\ 0 &{} 0 \end{array} \hspace{6pt} \xleftarrow {\varphi _3} \hspace{6pt} \emptyset \end{aligned}$$

## Symmetry Classes of QCPPs

### (Quasi)-symmetric QCPPs

Remember that a plane partition $\pi$ is quarter complementary inside a $(2a,2b,2c)$-box if for all $1 \le i \le 2a$ and $1 \le j \le 2b$, we have

3.1 $$\begin{aligned} \pi _{i,j} \cdot \pi _{2a+1-i,2b+1-j} = 0, \end{aligned}$$

and for $\pi _{i,j}>0$ exactly one of the following equations holds:

3.2 $$\begin{aligned} \pi _{i,j}+\pi _{2a+1-i,j}=2c, \qquad \text {or} \qquad \pi _{i,j}+\pi _{i,2b+1-j}=2c. \end{aligned}$$

Regarded as a regular plane partition, $\pi$ is called symmetric if $a=b$ and $\pi _{i,j}=\pi _{j,i}$ for all $1 \le i,j \le 2a$. By (3.1), we see that $\pi$ can only be symmetric if the entries on its anti-diagonal are 0, i.e. $\pi _{i,2a+1-i}=0$ for $1 \le i \le 2a$. Together with (3.2), this implies $\pi _{a,a}=2c$ and hence that the only symmetric quarter complementary plane partition is

$$\begin{aligned} \pi = \left( {\left\{ \begin{array}{ll} 2c \qquad &{}i,j \le a,\\ 0 &{} \text {otherwise,} \end{array}\right. } \right) _{1 \le i,j \le 2a}. \end{aligned}$$

In order to obtain more interesting objects, we omit the symmetry condition on the anti-diagonal and call a quarter complementary plane partition $\pi$ *quasi-symmetric* if $\pi _{i,j}=\pi _{j,i}$ for all $1 \le i,j \le 2a$ and $i \ne 2a+1-j$. Denote by ${{\,\textrm{QS}\,}}(a,c)$, the set of quarter complementary plane partitions inside a (2*a*, 2*a*, 2*c*)-box which are quasi-symmetric.

#### Proposition 3.1

Let *a*, *c* be positive integers. Then, ${{\,\textrm{QS}\,}}(a,c)$ is equal to

3.3 $$\begin{aligned} {{\,\textrm{QS}\,}}(a,c) = \varphi _1 \circ \varphi _2 \big ({{\,\textrm{QS}\,}}(a-1,c)\big ) {\dot{\cup }} \varphi _2 \circ \varphi _1 \big ({{\,\textrm{QS}\,}}(a-1,c)\big ) {\dot{\cup }} \varphi _3\big ({{\,\textrm{QS}\,}}(a,c-1)\big )\nonumber \\ \end{aligned}$$

#### Proof

Let $\pi \in {{\,\textrm{QS}\,}}(a,c)$. By Proposition 2.4, $\pi$ is either in $\varphi _3\big ({{\,\textrm{FCP}\,}}(a,a,c-1)\big )$,$\varphi _1\big ({{\,\textrm{FCP}\,}}(a-1,a,c)\big )$ or $\varphi _2\big ({{\,\textrm{FCP}\,}}(a,a-1,c)\big )$. In the first case, $\varphi _3^{-1}(\pi )$ is obviously quasi-symmetric and for each $\sigma \in {{\,\textrm{QS}\,}}(a,c-1)$, the partition $\varphi _3(\sigma )$ is also quasi-symmetric. Assume $\pi \in \varphi _1\big ({{\,\textrm{FCP}\,}}(a-1,a,c)\big )$. By the definition of $\varphi _1$, we have $\pi _{a,j}=\pi _{a+1,j}=c$ if $j \le a$ and $\pi _{a,j}=\pi _{a+1,j}=0$ if $j>a$. By the quasi-symmetry of $\pi$, this implies $\pi _{j,a}=\pi _{j,a+1}=c$ for $j < a$ and $\pi _{j,a}=\pi _{j,a+1}=0$ for $j > a+1$. Therefore, we obtain $\pi = \varphi _1\left( \varphi _2(\sigma ) \right)$ for $\sigma \in {{\,\textrm{FCP}\,}}(a-1,a-1,c)$. For $1 \le i,j \le 2(a-1)$ and $i+j \ne 2a-1$, we have

$$\begin{aligned} \sigma _{i,j}= \pi _{i,j} = \pi _{j,i} = \sigma _{j,i} \qquad&\text {for } i,j<a,\\ \sigma _{i,j}= \pi _{i,j+2} = \pi _{j+2,i} = \sigma _{j,i} \qquad&\text {for } i<a, j \ge a\\ \sigma _{i,j}= \pi _{i+2,j} = \pi _{j,i+2} = \sigma _{j,i} \qquad&\text {for } i \ge a, j <a,\\ \sigma _{i,j}= \pi _{i+2,j+2} = \pi _{j+2,i+2} = \sigma _{j,i} \qquad&\text {for } i,j \ge a,\\ \end{aligned}$$

where we used the definition of $\varphi _1$ and $\varphi _2$ for the first and last equality and the quasi-symmetry of $\pi$ for the second equality. This implies $\sigma \in {{\,\textrm{QS}\,}}(a-1,c)$. On the other hand if $\sigma \in {{\,\textrm{QS}\,}}(a-1,c)$, it follows by the same considerations that $\varphi _1\left( \varphi _2(\sigma ) \right)$ is quasi-symmetric and hence an element of ${{\,\textrm{QS}\,}}(a,c)$. The last case follows analogously. $\square$

By defining ${{\,\textrm{QS}\,}}(a,c)$ to consist of the “empty array” if either *a* or *c* is equal to 0, we obtain immediately the following corollary.

#### Corollary 3.2

The generating function for quasi-symmetric quarter complementary plane partitions is given by

$$\begin{aligned} \sum _{a,c > 0} |{{\,\textrm{QS}\,}}(a,c)|x^ay^c = \frac{(3- 2x-y)xy}{(1-x)(1-y)(1-2x-y)}. \end{aligned}$$

#### Proof

Using Proposition 3.1, we obtain

$$\begin{aligned} \sum _{a,c> 0} |{{\,\textrm{QS}\,}}(a,c)|x^ay^c{} & {} = \sum _{a,c> 0} \left( 2x|{{\,\textrm{QS}\,}}(a-1,c)|x^{a-1}y^c + y|{{\,\textrm{QS}\,}}(a,c-1)|x^{a}y^{c-1} \right) \\{} & {} = \sum _{a,c> 0} |{{\,\textrm{QS}\,}}(a,c)|x^ay^c (2x+y) + \sum _{c>0} 2x|{{\,\textrm{QS}\,}}(0,c)|y^c\\ {}{} & {} \quad + \sum _{a>0} y|{{\,\textrm{QS}\,}}(a,0)|x^a \\{} & {} = \sum _{a,c > 0} |{{\,\textrm{QS}\,}}(a,c)|x^ay^c (2x+y) + \frac{2xy}{1-y}+\frac{xy}{1-x}. \end{aligned}$$

We obtain the assertion by combining both sums over $a,c>0$ and factorising the expression. $\square$

### Cyclically Symmetric QCPPs

A plane partition $\pi$ is called *cyclically symmetric* if a point (*i*, *j*, *k*) in its Ferrers diagram implies that (*j*, *k*, *i*) is also in its Ferrers diagram. As we see next, there is no cyclically symmetric quarter complementary plane partition. Let $\pi$ be a cyclically symmetric quarter complementary Ferrers diagram inside a (2*a*, 2*a*, 2*a*)-box. It is not difficult to see, that being quarter complementary implies that one of the points (1, 1, 2*a*), (1, 2*a*, 1) or (2*a*, 1, 1) has to be part of $\pi$, and hence all of them since $\pi$ is cyclically symmetric. The set $\rho _{\{1,2\}}(\pi )$, therefore, contains the points (2*a*, 1, 1) and (1, 2*a*, 1) which is a contradiction to $\pi \cap \rho _{\{1,2\}}(\pi ) = \emptyset$.

Contrary to the previous subsection, we did not find an “interesting” generalisation of cyclically symmetric to “quasi-cyclically symmetric” for which we can deduce either a result or a conjecture in the case of QCPPs.

### Self- and Transpose-Complementary QCPPs

In order to study self- or transpose-complementary QCPPs, we need to specify the box we want to consider the complementation in. For a QCPP inside a (2*a*, 2*b*, 2*c*)-box, the possible boxes for complementation are a (2*a*, 2*b*, *c*)-, a (2*a*, *b*, 2*c*)- and an (*a*, 2*b*, 2*c*)-box. For symmetry reasons, it suffices to consider QCPPs which are self- or transpose-complementary inside a (2*a*, 2*b*, *c*)-box. We call a QCPP $\pi$ inside a (2*a*, 2*b*, 2*c*)-box *self-complementary* if $\pi _{i,j}+\pi _{2a+1-i,2b+1-j}=c$ for all $1 \le i \le 2a$ and $1 \le j \le 2b$, and *quasi-transpose-complementary*^2^ if $a=b$ and $\pi _{i,j}+\pi _{2a+1-j,2a+1-i}=c$ for all $1 \le i,j \le 2a$ with $i \ne 2a+1-j$. We obtain the following enumeration results.

#### Proposition 3.3

The number of self-complementary QCPPs inside a (2*a*, 2*b*, 2*c*)-box is $$\left( {\begin{array}{c}a+b\\ a\end{array}}\right) $$.

#### Proof

The condition $\pi _{i,j}+\pi _{2a+1-i,2b+1-j}=c$ implies that all entries are at most *c*. Together with (3.2) this implies that all entries are either 0 or *c*. Hence, by Proposition 2.4, $\pi$ is either of the form $\varphi _1(\sigma )$ or $\varphi _2(\sigma )$ for an appropriate QCPP $\sigma$. For $\pi =\varphi _1(\sigma )$ and $1 \le i \le (a-1)$ and $1 \le j \le 2b$, we have

$$\begin{aligned} \sigma _{i,j}+\sigma _{2a-1-i,2b+1-j}= \pi _{i,j}+\pi _{2a+1-i,2b+1-j}=c, \end{aligned}$$

i.e. $\sigma$ is self-complementary inside a $(2(a-1),2b,2c)$-box. On the other hand, for each self-complementary $\sigma$ inside a $(2(a-1),2b,2c)$-box the QCPP $\varphi _1(\sigma )$ is self-complementary inside a (2*a*, 2*b*, 2*c*)-box. The case $\pi =\varphi _2(\sigma )$ follows analogously. The assertion is now immediate by induction on $a+b$. $\square$

#### Proposition 3.4

A QCPP inside a (2*a*, 2*a*, 2*c*)-box is quasi-transpose-complementary if and only if it is quasi-symmetric and self-complementary. The number of quasi-transpose-complementary QCPPs inside a (2*a*, 2*a*, 2*c*)-box is $2^{a}$.

#### Proof

The condition $\pi _{i,j}+\pi _{2a+1-j,2a+1-i}=c$ together with (3.2) implies that all entries of $\pi$ are either 0 or *c*. Since $\pi$ is quarter complementary inside a (2*a*, 2*a*, 2*c*)-box, the sum over all entries must be $2 a^2c$. Hence, exactly $2a^2$ entries are equal to *c* and $2a^2$ entries are equal to 0. For $i \le a$ exactly one of the equations $\pi _{i,i}+\pi _{i,2a+1-i}=2c$ or $\pi _{i,i}+\pi _{2a+1-i,i}=2c$ holds by (2.3). Hence, exactly half of the entries on the anti-diagonal are equal to *c*. For $i \ne 2a+1-j$ we, therefore, have $\pi _{2a+1-j,2a+1-i}=c$ exactly if $\pi _{i,j}=0$, which is by (3.1) equivalent to $\pi _{2a+1-i,2a+1-j}=c$. This implies that $\pi$ is quasi-symmetric and, therefore, also self-complementary. It is immediate that a quasi-symmetric, self-complementary QCPP is also quasi-transpose-complementary.

By the proof of Proposition 3.1 and Proposition 3.3 each quasi-transpose-complementary QCPP $\pi$ is of the form $\varphi _1 \circ \varphi _2 (\sigma )$ or $\varphi _2 \circ \varphi _1 (\sigma )$, where $\sigma$ is a quasi-transpose-complementary QCPP inside a $\big (2(a-1),2(a-1),2c\big )$-box. The assertion follows now by induction on *a*. $\square$

## Quasi-symmetry Classes of Plane Partitions

### Three Conjectures

In the previous section we introduced variations of two symmetry classes for quarter complementary plane partitions. The aim of this section is to consider these and similar symmetry classes for plane partitions. The following three conjectures were found by computer experiments. We have added the according data as well as explicit guessed enumeration formulas for small values of one of the parameters in the appendix.

#### Conjecture 4.1

Let us denote by ${{\,\textrm{qspp}\,}}(a,c)$ the number of quasi-symmetric plane partitions inside an (*a*, *a*, *c*)-box. Then,

4.1 $$\begin{aligned} {{\,\textrm{qspp}\,}}(a,c-a) ={\left\{ \begin{array}{ll} c \left( {\begin{array}{c}c+a-1\\ 2a-1\end{array}}\right) p_a(c) \qquad &{} a \text { is even},\\ \left( {\begin{array}{c}c+a-1\\ 2a-1\end{array}}\right) p_a(c) \qquad &{} a \text { is odd},\\ \end{array}\right. } \end{aligned}$$

where $p_a(c)$ is an irreducible polynomial in ${\mathbb {Q}}[c]$ that is even, i.e. $p_a(c)=p_a(-c)$. Further the common denominator of the coefficients of $p_a(c)$ is a product of “small primes”.

#### Conjecture 4.2

We call a plane partition $\pi$ inside an (*a*, *a*, 2*c*)-box *quasi-transpose complementary of second kind* (QTC2), if $\pi$ is transpose-complementary except along the diagonal, i.e. $\pi _{i,j}+\pi _{a+1-j,a+1-i}=c$ for all $1 \le i,j \le a$ with $i \ne j$. Then, for $a\ge 2$, the number ${{\,\textrm{qtcpp}\,}}_2(a,c)$ of QTC2 plane partitions inside an (*a*, *a*, 2*c*)-box is given by

4.2 $$\begin{aligned} {{\,\textrm{qtcpp}\,}}_2\left( a,c-\frac{a}{2}\right) =c \left( {\begin{array}{c}c+\frac{a}{2}-1\\ a-1\end{array}}\right) p_a(c), \end{aligned}$$

where $p_a(c)$ is an irreducible polynomial in ${\mathbb {Q}}[c]$ that is even. Further the common denominator of the coefficients of $p_a(c)$ is a product of “small primes”.

#### Conjecture 4.3

Denote by ${{\,\textrm{qtcspp}\,}}_2(a,c)$ the number of symmetric QTC2 plane partitions. Then, for $a\ge 2$,

4.3 $$\begin{aligned} {{\,\textrm{qtcspp}\,}}_2\left( a,c-\frac{a}{2}\right) = {\left\{ \begin{array}{ll} \left( {\begin{array}{c}c+\frac{a}{2}-1\\ a-1\end{array}}\right) p_a(c) \qquad &{} a \equiv 3 \text { modulo} \ 4, \\ c\left( {\begin{array}{c}c+\frac{a}{2}-1\\ a-1\end{array}}\right) p_a(c) \qquad &{} \text {otherwise},\\ \end{array}\right. } \end{aligned}$$

where $p_a(c)$ is a polynomial in ${\mathbb {Q}}[c]$ that is even and for *a* even irreducible in ${\mathbb {Q}}[x]$. Further the common denominator of the coefficients of $p_a(c)$ is a product of “small primes”.

### Proof of Theorem 1.2

Let $\pi$ be a quasi-transpose-complementary plane partition (QTCPP) inside an (*n*, *n*, *c*)-box. By definition, we have $\pi _{n-j,j} \ge \pi _{n-j+1,j} \ge \pi _{n-j+1,j+1}=c-\pi _{n-j,j}$ for each $1 \le j \le n-1$ and equivalently by multiplying the above inequalities by $-1$ and adding *c* to it, $\pi _{n-j+1,j+1}=c-\pi _{n-j,j} \le c-\pi _{n-j+1,j} \le \pi _{n-j,j}$. Hence, $\pi$ stays a QTCPP if we replace its diagonal entries $\pi _{n-j+1,j}$ by $\max \left( \pi _{n-j+1,j},c-\pi _{n-j+1,j} \right)$ for all $1 \le j \le n$. We denote the resulting QTCPP by ${\widehat{\pi }}$. Denote by $d({\widehat{\pi }})$ the number of anti-diagonal entries which are equal to ${\widehat{c}}=\left\lfloor \frac{c}{2} \right\rfloor$ and define the weight $\omega ({\widehat{\pi }})$ as

$$\begin{aligned} \omega ({\widehat{\pi }}) = {\left\{ \begin{array}{ll} 2^n \qquad &{} c\text { is odd,}\\ 2^{n-d({\widehat{\pi }})} &{} c\text { is even}. \end{array}\right. } \end{aligned}$$

Since $|\{\pi _{n-j+1,j},c-\pi _{n-j+1,j}\}|=1$ implies that *c* is even and $\pi _{n-j+1,j}={\widehat{c}}$, it is clear that there are $\omega ({\widehat{\pi }})$ many QTCPPs mapping to ${\widehat{\pi }}$ by the above map. Hence, the number of QTCPPs is equal to the weighted enumeration of QTCPPs ${\widehat{\pi }}$ whose anti-diagonal entries are at least $\frac{c}{2}$. In the following, we present two (and a half) proofs for the weighted enumeration of these ${\widehat{\pi }}$.

**Fig. 3:**
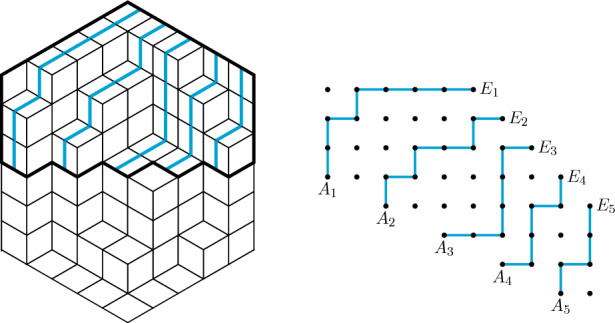
A QTCPP ${\widehat{\pi }}$ inside a (5, 5, 6)-box whose anti-diagonal entries are at least 3 (left) and its corresponding lattice path configuration (right)

For odd *c*, each ${\widehat{\pi }}$ corresponds to a plane partition with entries at most ${\widehat{c}}$ for which the *i*-th row from top has at most $n+1-i$ positive entries. The number of these plane partitions can be found in [16, Corollary 4.1], compare also with [4, Corollary 4.1]. For even *c*, each ${\widehat{\pi }}$ corresponds to a lozenge tiling in the “top half” of an hexagon with side lengths *n*, *n*, *c*, *n*, *n*, *c*, where the bottom of the region is a zig-zag shape directly below the centre line of the hexagon, and each 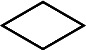 lozenge at the bottom of the region is weighted by $\frac{1}{2}$, see Fig. 3 (right) for an example. The number of these tilings is given in [4, Corollary 4.3]. The assertion follows in both cases using the explicit formulas from [4, 16].

For a second proof of Theorem 1.2, denote by ${{\,\textrm{qtcpp}\,}}(n,n,c)$ the number of QTCPPs inside an (*n*, *n*, *c*)-box and denote by *M*(*R*) the number of perfect matchings of a region *R*. We can rephrase the above observations as

$$\begin{aligned} {{\,\textrm{qtcpp}\,}}(n,n,2c) = 2^n M(P^\prime _{n,n,c}), \qquad {{\,\textrm{qtcpp}\,}}(n,n,2c+1) = 2^n M(P_{n,n,c}), \end{aligned}$$

where the region $P_{n,n,c}$ is defined in [4, Sect. 4, Fig. 5] and $P^\prime _{n,n,c}$ is defined in [4, Sect. 4, Fig. 10]. On the one side, we have by Ciucu’s factorization theorem for graphs with reflective symmetry [3] the identity

$$\begin{aligned} {{\,\textrm{PP}\,}}(n,n,2c) = 2^n M(P^\prime _{n,n,c}) M(P_{n-1,n-1,c}), \end{aligned}$$

where ${{\,\textrm{PP}\,}}(n,n,2c)$ denotes the number of plane partitions inside an (*n*, *n*, 2*c*)-box (compare for example with [3] or [4, Eq. (4.16)]). This implies the assertion for even *c* if we know it for odd *c* and vice versa using the explicit formulas for the number of (symmetric) plane partitions. Further, it is well known, that $2^n M(P^\prime _{n,n,c})=SPP(n,n,2c)$, see [5, Eq. (5.1)] or [6, Eq. (2.6)] and that $M(P_{n-1,n-1,c}) = {{\,\textrm{TCPP}\,}}(n,n,2c)$, where ${{\,\textrm{TCPP}\,}}(n,n,2c)$ is the number of transpose-complementary plane partitions inside an (*n*, *n*, 2*c*)-box (see for example [3, Sect. 6]). Combining the above and the assertion already proved above, we obtain immediately the numerical connection between SPPs and TCPPs stated in (1.5).

Finally, we present another proof using non-intersecting lattice paths. We regard ${\widehat{\pi }}$ as a lozenge tiling as above and draw *n* lattice paths ending at the top right boundary of the hexagon in the following way. The allowed steps for the paths are 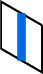 and 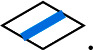 Finally, the *i*-th path from left has length $n+1-i+{\widehat{c}}$; see Fig. 3 (left) for an example. By straightening the paths, we obtain non-intersecting lattice paths starting at $A_i=(2i,-i)$ and ending at $E_i=(n+1+i,{\widehat{c}}-i)$ with north-steps (0, 1) and east-steps (1, 0), see Fig. 3 (right). For odd *c*, each family of paths has the same weight, namely $2^n$. Hence, the weighted enumeration is, therefore, by the Lindström–Gessel–Viennot Theorem equal to

4.4 $$\begin{aligned} 2^n\det _{1 \le i,j \le n} \left( \left( {\begin{array}{c}n+{\widehat{c}}+1-i\\ n+1+j-2i\end{array}}\right) \right) . \end{aligned}$$

For even *c*, we see that an anti-diagonal entry $\pi _{n+1-j,j}={\widehat{c}}$ corresponds to the *j*-th path starting with an east-step. Define the points $B^0_i=(2i+1,-i)$ and $B^1_i=(2i,-i+1)$ which are reached from $A_i$ by an east-step or a north-step, respectively. By deleting the first step of each path, we obtain a family of non-intersecting lattice paths starting from either $B^0_i$ or $B^1_i$, where the weight is given by 2 to the power of the number of times we start at $B^1_i$. Using again the Lindström–Gessel–Viennot Theorem, we obtain for the weighted enumeration

4.5 $$\begin{aligned}{} & {} \sum _{(b_1,\ldots ,b_n) \in \{0,1\}^n}\det _{1 \le i,j \le n} \left( 2^{b_i}\left( {\begin{array}{c}n+{\widehat{c}}-i\\ n+j-2i+b_i\end{array}}\right) \right) \nonumber \\{} & {} \quad = \det _{1 \le i,j \le n} \left( \left( {\begin{array}{c}n+{\widehat{c}}-i\\ n+j-2i\end{array}}\right) +2\left( {\begin{array}{c}n+{\widehat{c}}-i\\ n+j-2i+1\end{array}}\right) \right) , \end{aligned}$$

where we used the multilinearity of the determinant in the last step. Both determinants could be evaluated by guessing the corresponding LU decomposition and using the Pfaff–Saalschütz-summation formula, see for example [17, Eq. (2,3,1,3); Appendix (III.2)]; we omit, however, the details since there is a simpler and more elegant solution as follows. First, we rewrite the determinant in (4.5) as

4.6 $$\begin{aligned}{} & {} \det _{1 \le i, j \le n} \left( \left( {\begin{array}{c}n+{\widehat{c}}-i+1\\ n+j-2i+1\end{array}}\right) \frac{2{\widehat{c}}+n-j+1}{{\widehat{c}}+n-i+1} \right) \nonumber \\{} & {} \quad =\det _{1 \le i, j \le n} \left( \left( {\begin{array}{c}n+{\widehat{c}}-i+1\\ n+j-2i+1\end{array}}\right) \right) \prod _{i=1}^n \frac{2{\widehat{c}}+n-i+1}{{\widehat{c}}+n-i+1}. \end{aligned}$$

Then, both determinants are special cases of determinant evaluation [10, Eq. (3.13)]

$$\begin{aligned} \det _{1 \le i,j \le n}\left( \left( {\begin{array}{c}B L_i+A\\ L_i+j\end{array}}\right) \right)= & {} \frac{\prod \nolimits _{1 \le i < j \le n}(L_i-L_j)}{\prod \nolimits _{i=1}^n (L_i+n)!}\prod _{i=1}^n\frac{(B L_i+A)!}{((B-1)L_i+A-1)!}\\{} & {} \prod _{i=1}^n(A-Bi+1)_{i-1}, \end{aligned}$$

by setting $L_i=n+1-2i$, $B=\frac{1}{2}$ and $A={\widehat{c}}+\frac{n+1}{2}$.

## Appendix A. Data from the Computer Experiments

The data presented in the appendix was obtained by generating the according objects using Mathematica; the code can be obtained on the authors webpage https://homepage.univie.ac.at/florian.schreier-aigner/data/data_generation.nb.

### A.1 Quasi-symmetric Plane Partitions

The values for the number ${{\,\textrm{qspp}\,}}(a,c)$ of quasi-symmetric plane partitions inside an (*a*, *a*, *c*)-box with $a \le 6$ and $c \le 10$ are shown in the next table.

**Table:**

*c* \ *a*	1	2	3	4	5	6
0	1	1	1	1	1	1
1	2	6	12	32	64	164
2	3	20	69	400	1442	7952
3	4	50	272	3052	18,544	200,956
4	5	105	846	16,932	164,686	3,284,589
5	6	196	2232	74,868	1,118,080	38,963,092
6	7	336	5214	278,928	6,178,097	360,346,984
7	8	540	11,088	908,336	28,977,472	2,727,638,524
8	9	825	21,879	2,653,001	118,868,458	17,499,041,992
9	10	1210	40,612	7,081,776	435,998,528	97,667,820,784
10	11	1716	71,643	17,524,416	1,454,331,440	483,901,238,656

Using the above values, we conjecture the following formulas for ${{\,\textrm{qspp}\,}}(a,c)$ for $1 \le a \le 6$:

$$\begin{aligned} {{\,\textrm{qspp}\,}}(1,c-1)&= \left( {\begin{array}{c}c\\ 1\end{array}}\right) ,\\ {{\,\textrm{qspp}\,}}(2,c-2)&= c \left( {\begin{array}{c}c+1\\ 3\end{array}}\right) \frac{1}{2} ,\\ {{\,\textrm{qspp}\,}}(3,c-3)&= \left( {\begin{array}{c}c+2\\ 5\end{array}}\right) \frac{c^2-2}{7},\\ {{\,\textrm{qspp}\,}}(4,c-4)&= c \left( {\begin{array}{c}c+3\\ 7\end{array}}\right) \frac{41 c^4-229 c^2-892}{23760},\\ {{\,\textrm{qspp}\,}}(5,c-5)&= \left( {\begin{array}{c}c+4\\ 9\end{array}}\right) \frac{202 c^8 -3137 c^6+5563 c^4-123588 c^2+352800}{30630600},\\ {{\,\textrm{qspp}\,}}(6,c-6)&= c \left( {\begin{array}{c}c+5\\ 11\end{array}}\right) \frac{1}{161911881331200}\\&\qquad \left( 56381 c^{12}- 1850347 c^{10} + 11282865 c^8\right. \\&\quad \left. - 28759181 c^6 - 1859025278 c^4 + 20697349128 c^2 + 194655992832\right) . \end{aligned}$$

### A.2. Quasi-transpose-Complementary Plane Partitions of Second Kind

For the number ${{\,\textrm{qtcpp}\,}}_2(a,c)$ of QTC2 plane partitions inside an (*a*, *a*, *c*)-box with $a \le 6$ and $c\le 10$, we have the following values:

**Table:**

*c* \ *a*	1	2	3	4	5	6
0	1	1	1	1	1	1
1	3	4	9	24	70	216
2	5	9	42	224	1578	12,177
3	7	16	138	1280	19,157	314,624
4	9	25	363	5361	155,270	4,860,048
5	11	36	819	18,088	943,008	51,955,744
6	13	49	1652	52,032	4,606,320	420,545,536
7	15	64	3060	132,408	18,969,942	2,735,918,368
8	17	81	5301	305,745	68,084,583	14,918,043,569
9	19	100	8701	652,432	218,198,470	70,303,307,672
10	21	121	13,662	1,304,160	635,987,530	293,079,258,017

We conjecture the following explicit formulas for ${{\,\textrm{qtcpp}\,}}_2(a,c)$ for $1 \le a \le 6$:

$$\begin{aligned} {{\,\textrm{qtcpp}\,}}_2\left( 1,c-\frac{1}{2}\right)= & {} 2c,\\ {{\,\textrm{qtcpp}\,}}_2(2,c-1)= & {} c\left( {\begin{array}{c}c\\ 1\end{array}}\right) ,\\ {{\,\textrm{qtcpp}\,}}_2\left( 3,c-\frac{3}{2}\right)= & {} c\left( {\begin{array}{c}c+\frac{1}{2}\\ 2\end{array}}\right) \frac{4c^2+11}{30},\\ {{\,\textrm{qtcpp}\,}}_2(4,c-2)= & {} c\left( {\begin{array}{c}c+1\\ 3\end{array}}\right) \frac{5c^4+19c^2-16}{280},\\ {{\,\textrm{qtcpp}\,}}_2\left( 5,c-\frac{5}{2}\right)= & {} c\left( {\begin{array}{c}c+\frac{3}{2}\\ 4\end{array}}\right) \\{} & {} \frac{35584 c^8+ 586496 c^6- 66144 c^4 - 2346256 c^2 +4267795}{461260800},\\ {{\,\textrm{qtcpp}\,}}_2(6,c-3)= & {} c\left( {\begin{array}{c}c+2\\ 5\end{array}}\right) \frac{1}{256505356800}\\{} & {} \left( 73325 c^{12} +1648357 c^{10} -12312285 c^8 + 29029591 c^6 \right. \\{} & {} \left. + 201378740 c^4- 876526848 c^2 + 395435520\right) . \end{aligned}$$

### A.3. Quasi-transpose-Complementary Symmetric Plane Partitions of Second Kind

Computer experiments yield the following values for the number ${{\,\textrm{qtcspp}\,}}_2(a,c)$ of symmetric QTC2 plane partitions inside an (*a*, *a*, *c*)-box with $a \le 6$ and $c \le 10$.

**Table:**

*c* \ *a*	1	2	3	4	5	6
0	1	1	1	1	1	1
1	3	4	7	12	22	40
2	5	9	26	68	210	625
3	7	16	70	260	1265	5728
4	9	25	155	777	5642	36,876
5	11	36	301	1960	20,328	184,224
6	13	49	532	4368	62,424	759,708
7	15	64	876	8856	169,290	2,695,200
8	17	81	1365	16,665	415,635	8,468,889
9	19	100	2035	29,524	940,654	24,078,184
10	21	121	2926	49,764	1,989,130	62,949,289

For the first few values of *a*, we conjecture the following formulas for ${{\,\textrm{qtcspp}\,}}_2(a,c)$:

$$\begin{aligned} {{\,\textrm{qtcspp}\,}}_2\left( 1,c-\frac{1}{2}\right)= & {} 2c,\\ {{\,\textrm{qtcspp}\,}}_2(2,c-1)= & {} c\left( {\begin{array}{c}c\\ 1\end{array}}\right) ,\\ {{\,\textrm{qtcspp}\,}}_2\left( 3,c-\frac{3}{2}\right)= & {} \left( {\begin{array}{c}c+\frac{1}{2}\\ 2\end{array}}\right) \frac{4c^2+3}{12},\\ {{\,\textrm{qtcspp}\,}}_2(4,c-2)= & {} c\left( {\begin{array}{c}c+1\\ 3\end{array}}\right) \frac{c^2+1}{10},\\ {{\,\textrm{qtcspp}\,}}_2\left( 5,c-\frac{5}{2}\right)= & {} c\left( {\begin{array}{c}c+\frac{3}{2}\\ 4\end{array}}\right) \left( {\begin{array}{c}c+\frac{1}{2}\\ 2\end{array}}\right) \frac{4 c^4+17}{315},\\ {{\,\textrm{qtcspp}\,}}_2(6,c-3)= & {} c\left( {\begin{array}{c}c+2\\ 5\end{array}}\right) \frac{3c^6+15c^4-58c^2+200}{9240},\\ {{\,\textrm{qtcspp}\,}}_2\left( 7,c-\frac{7}{2}\right)= & {} \left( {\begin{array}{c}c+\frac{5}{2}\\ 6\end{array}}\right) \left( {\begin{array}{c}c+\frac{1}{2}\\ 2\end{array}}\right) \frac{1}{15498362880}\\{} & {} \left( 66816 c^8 + 798464 c^6 - 4402464 x^4 \right. \\{} & {} \left. +34239856x^2-148134987\right) ,\\ {{\,\textrm{qtcspp}\,}}_2(9,c-\frac{9}{2})= & {} c\left( {\begin{array}{c}c+\frac{7}{2}\\ 8\end{array}}\right) \frac{1}{1750824414062051328000}\\{} & {} \left( 9387442176 c^{16} + 242529468416 c^{14}\right. \\{} & {} -6409671229440 c^{12}+148966135521280 c^{10} \\{} & {} -2812830240332288 c^8 +29468432544824832 c^6 \\{} & {} - 130634547937730368 c^4 + 73031174580058272 c^2 \\{} & {} \left. +97863783090792345 \right) . \end{aligned}$$

## Footnotes

1 Formally, we have for each ${\textbf{n}}=(n_1,\ldots ,n_{d+1})$ a different map $\varphi _k$. However, since ${\textbf{n}}$ will always be clear from the context, we do not include ${\textbf{n}}$ in the notation.

2 It is immediate that there are no transpose-complementary QCPPs since the condition $\pi _{i,j}+\pi _{2a+1-j,2a+1-i}=c$ on the anti-diagonal contradicts (2.2). Similar to quasi-symmetric, we, therefore, exclude the condition on the anti-diagonal to obtain interesting objects.
